# Anorexia and Young Womens’ Personal Networks: Size, Structure, and Kinship

**DOI:** 10.3389/fpsyg.2022.848774

**Published:** 2022-04-19

**Authors:** Oxana Mikhaylova, Sofia Dokuka

**Affiliations:** Institute of Education, HSE University, Moscow, Russia

**Keywords:** anorexia, networks, personal networks, mental health, young women

## Abstract

Anorexia is a serious threat to young women’s wellbeing worldwide. The effectiveness of mental health intervention and treatment is often evaluated on the basis of changes in the personal networks; however, the development of such measures for young women with anorexia is constrained due to the lack of quantitative descriptions of their social networks. We aim to fill this substantial gap. In this paper, we identify the basic properties of these women’s personal networks such as size, structure, and proportion of kin connections. The empirical analysis, using a concentric circles methodology, is based on 50 ego networks constructed on data drawn from interviews with Russian-speaking bloggers who have been diagnosed with anorexia and write about this condition. We conclude that young women with anorexia tend to support a limited number of social ties; they are prone to select women as alters, but do not have a preference to connect to their relatives. Further research is needed to elucidate whether these personal network characteristics are similar among women with anorexia who belong to different age, ethnic, cultural, and income groups.

## Introduction

Anorexia is an eating disorder (ED) related to significant physical and mental health problems in adolescence and adulthood (Galmiche et al., 2019). This ED is characterized by restriction of food intake, fear of becoming fatter, and distortions of body image (American Psychiatric Association, 2013). Girls are 2–3 times more likely than boys to face anorexia in their teenage years (Steiner and Lock, 1998; Hoek, 2006; Nagl et al., 2016; Galmiche et al., 2019). This is often explained by the social expectations of women (especially women’s bodies) in contemporary societies (Boskind-Lodahl, 1976; Mahowald, 1992; Malson, 1998; Bruch, 2001; Dahlenburg et al., 2019; Giordano, 2020).

Anorexia, as with other self-harming behaviors, is associated with difficulties in interpersonal relations (Tiller et al., 1997; Patel et al., 2016; Cardi et al., 2018; Pace et al., 2018; Birmachu et al., 2019; Okada et al., 2019). Although the relationship between anorexia and personal connections has been investigated, research on this topic has mostly provided qualitative descriptions or only indirectly focused on young women’s social networks (Leonidas and dos Santos, 2014). Studies that have revealed quantitative features of the personal networks of young women with anorexia are scarce and were conducted in a limited number of countries (Quiles Marcos and Terol Cantero, 2009; Tubaro and Mounier, 2014; Pallotti et al., 2018). Additionally, we should note the lack of research on the social networks of people with anorexia in Eastern Europe, especially Russia. Therefore, further research is needed to shed light on the social contexts of these women.

The present study fills this gap. We describe the main characteristics of the personal networks of adolescents and young adult women with diagnosed anorexia. The analysis is based on self-reported ego-networks collected from 50 Russian-speaking young women in the summer (July–August) of 2020.

The contribution of our study is twofold. First, we provide quantitative descriptions of personal networks of young women with anorexia. There is a lack of information on such networks in the scientific literature and our paper provides this valuable data. Second, such information has practical significance because the measurement of the effectiveness of the many types of interventions and treatments targeted at the improvement of women’s mental health is often based on the analysis of changes in their personal networks (Anderson et al., 2015; Siette et al., 2017; Ma et al., 2020).

The paper starts with an overview of network-based research on people with anorexia. It proceeds with a description of the methodology. Finally, the results are reported and discussed.

## Literature Review

During adolescence, teenagers actively transform their personal social networks along with significant life events such as separating from their families, taking their first steps in their educational and professional development, and establishing new friends and romantic contacts (Giordano, 2003; Cotterell, 2013). People who surround young adults and adolescents at this time could either provide support and contribute to their wellbeing in various social spheres or influence their involvement in risky and practices deemed deviant by society (López et al., 1989; Wells and Rankin, 1991; Lorant and Tranmer, 2019; Webster et al., 2021).

Anorexia is one of the self-harming behaviors that often originates in adolescence and is most prevalent among girls (Galmiche et al., 2019). Moreover, it is associated with the highest rates of premature mortality among EDs (Arcelus et al., 2011; Keshaviah et al., 2014) and is connected with multiple comorbid mental health problems (Jordan et al., 2008; Martinussen et al., 2017; Marucci et al., 2018). Adult food intake and body image are both significantly dependent on the social networks that are formed in adolescence (Ali and Lindström, 2006; Fletcher et al., 2011; Simone et al., 2018; Grund and Tatum, 2019). For example, in social interactions within female peer groups during adolescence, women may acquire cultural values of beauty and practices of body care *via* the emulation of dieting practices, body size comparisons, and weight-based teasing that encourages them to lose weight (Allison et al., 2014). All in all, the effect of personal social networks turns out to be an important factor in behavior deemed deviant by society. Therefore, the quantitative analysis of personal networks of young women with anorexia could have significant implications for the prevention and management of this ED (Ferguson et al., 2011).

### Network Characteristics of People With Anorexia

The psychological and sociological literature suggests that people with anorexia might experience difficulties with communication, feel socially isolated, and report lower levels of social support from family members and significant others (Tiller et al., 1997; Levine, 2012; Patel et al., 2016; Cardi et al., 2018; Pace et al., 2018; Birmachu et al., 2019; Okada et al., 2019). Specifically, they are described as having problems establishing new relationships and maintaining old ones, caused by a distrust of others (Patel et al., 2016; Cardi et al., 2018; Quiles et al., 2020; Datta et al., 2021; Rowlands et al., 2021). These difficulties are experienced both before and after anorexia. Some scholars argue that these problems usually become more severe during anorexia (Cardi et al., 2018). Although the interpersonal relationships of people with anorexia have been described in theoretical and empirical qualitative studies (Patel et al., 2016; Westwood et al., 2016; Paul et al., 2018), quantitative descriptions of these people’s personal networks are scarce (Leonidas and dos Santos, 2014). This, consequently, significantly restricts perspectives for the exploration of social network factors in anorexia development.

To extend our knowledge of the structural characteristics of the personal networks of people with anorexia, we will focus on three interrelated aspects: (1) *network size*, (2) *network structure*, (3) *kinship network.* In this section of the paper, along with a literature review, we introduce our hypotheses and describe the characteristics of social networks.

#### Network Size

Network size, one of the central characteristics of the social network structure, is usually defined as the number of alters in a personal social network (Wasserman and Faust, 1994; Crawford et al., 2018). Literature on social support reports that the average size of the social support network of individuals with anorexia varies from 5 to 16 alters (Quiles Marcos and Terol Cantero, 2009; Tubaro and Mounier, 2014; Pallotti et al., 2018). Notably, two of these three studies include both men and women (Tubaro and Mounier, 2014; Pallotti et al., 2018), making it difficult to hypothesize whether the network size of people with diagnosed anorexia is gender-specific. Therefore, based on previous studies of mixed-gendered samples we hypothesize that: *The average network size of young women with anorexia will range from 5 to 16 actors (H1).*

#### Network Cohesion

Social cohesion is a widely applied concept in the social sciences (Friedkin, 2004). It might be defined as a resource of a group or society that affects individuals at both local and community levels (Lin, 2001; Martí et al., 2017). From a social network perspective, cohesion usually refers to the general level of network connectedness (Wasserman and Faust, 1994; Martí et al., 2017). In order to measure social cohesion, researchers usually calculate the density of the social network (Pallotti et al., 2018). In this paper, we consider network cohesion from a different perspective. We argue that the local sub-group structure within personal networks needs to be taken into account, because the extent to which an actor is connected to multiple cohesive subsets of alters plays a significant role in certain types of social support (Martí et al., 2017). Previous research demonstrates that individuals within the personal networks of people with anorexia are at least acquainted with each other or maintain other types of relationships such as friendship (Tubaro and Mounier, 2014; Pallotti et al., 2018). Thus, as a measure of social cohesion we use the proportion of isolates in an individual’s social network and based on previous empirical findings (Tubaro and Mounier, 2014; Pallotti et al., 2018), we hypothesize that: *The network structure of young women with anorexia will be of middle-high cohesiveness. This means that the proportion of isolates in the networks would be less than 0.5 (H2)*.

#### Kinship Network

People with anorexia tend to have difficulties with emancipation from their families and are prone to having limited social contact with people outside their families (Ruuska et al., 2007). Moreover, they frequently mention their mothers among their primary social support providers (Quiles Marcos and Terol Cantero, 2009). However, scholars indicate that the social surroundings of people with anorexia have become more diverse due to technological advances, specifically the opportunity to establish and maintain relationships *via* the internet, and include a large number of partners and friends (Tubaro and Mounier, 2014; Pallotti et al., 2018). Some papers demonstrate that these connections may worsen their health condition because the members of these communities can motivate each other toward extreme weight loss, as in pro-ana communities, which treat anorexia as a manageable lifestyle (Rodgers et al., 2012; Mento et al., 2021; Osler and Krueger, 2021; Nova et al., 2022). At the same time, online communities comprising people with anorexia who support personal recovery exist, such as pro-recovery communities that encourage people with anorexia to get treatment and may help them improve their health (Branley and Covey, 2017; Lamarre and Rice, 2017; Kenny et al., 2019). Additionally, the personal networks of people with anorexia might include health workers such as psychologists or psychiatrists (Quiles Marcos and Terol Cantero, 2009; Tubaro and Mounier, 2014; Pallotti et al., 2018). Summarizing, current research suggests that kin would outnumber non-kin in personal social networks of people with anorexia, although the proportion of non-kin might increase for various reasons. This leads us to our third hypothesis: *Among the members of the young women with anorexia, kin will outnumber non-kin (H3).*

People with anorexia usually state that their body condition and weight management are the most important parts of their life, both after having anorexia and during its active phase, and scholars claim that people outside the family circle provide people with anorexia with dieting tips and related information; for example, friends might give advice on pills and exercise (Brotsky and Giles, 2007; Haas et al., 2011; Pallotti et al., 2018). While family members do not always share their attitudes toward food and body, we suppose that alters outside the family circle could be classified by people with anorexia as more significant to them than family members because of their potential function as providers of body management information. We therefore hypothesize that: *The non-kin members of the social networks of young women with anorexia will be perceived subjectively as being closer (H4).*

Finally, as the social networks of people with anorexia are usually predominantly composed of females (Tubaro and Mounier, 2014; Pallotti et al., 2018) we hypothesized that: *Of the non-kin personal network members of women with anorexia, a majority will be women (H5).*

Additionally, in the section “Results,” we report the basic descriptive structural metrics of personal networks of women with anorexia. These descriptions contribute to the general understanding of the composition of the social networks of people with anorexia.

## Materials and Methods

### Sample

We collected data from young Russian-speaking female bloggers who have public blogs on the Russian social networking site VK.^1^ In these blogs, these young women write about their current health status and relationships with other people as well as other personal information. Additionally, bloggers interact with their audience *via* text and video on anorexia-related topics. The participants in our study were recruited using purposive sampling (Onwuegbuzie and Leech, 2015) to ensure the heterogeneity of narratives. Namely, we created a list of women who might be willing to participate in our research with a quota on the city and the number of subscribers on VK, then we contacted these people in personal text messages. Out of 156 women contacted, 50 agreed to participate in this study. As a result, in July and August 2020 we conducted biographical interviews with young female bloggers medically diagnosed with anorexia from more than 30 different Russian, Ukrainian, Kazakhstani, and Belarussian cities. Some of these bloggers hold pro-anorexia views (35), while the others support recovery (15). The age of participants ranges from 14 to 25. The description of the sample is in the Supplementary Appendix Table 1. The interviews were conducted *via* Skype due to the quarantine measures in Russia.

### Data Collection

**FIGURE 1:**
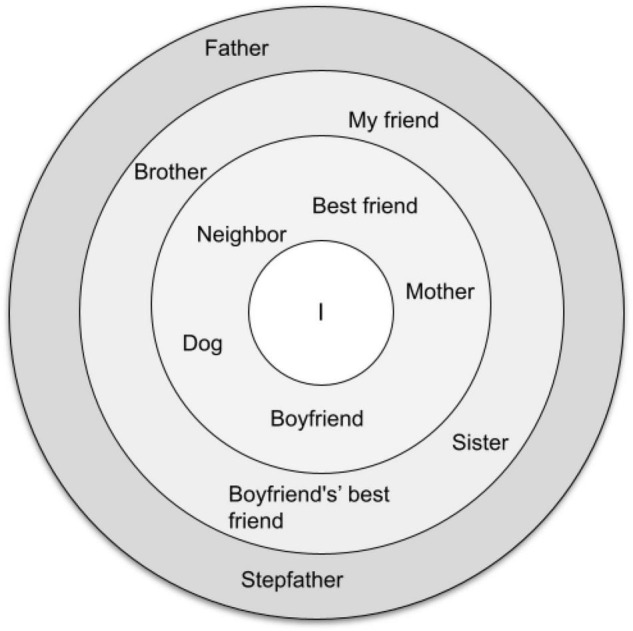
The social contacts map of participant 2 (translated from Russian). In the center of the map is the blogger herself. In the first circle are people and an animal who she named the most significant to her (her dog, boyfriend, mother, neighbor, and best friend). In the second circle are people she claimed to be less important (brother, friend, sister, and boyfriend’s best friend). The least close to the participant are her father and stepfather. These people are placed in the third circle. She placed no one outside the circles, i.e., this participant did not name people who are not important to her.

We gathered the data on the personal network connections of these young women using semi-structured biographical interviews (Rosenthal, 2004). The interviews consisted of the following parts: (1) The unstructured narratives of the young women about their lives from the very beginning to the time of the interview, (2) Clarifying questions, and (3) The elicitation of their personal social networks. The personal networks were obtained and analyzed using the concentric circles method (Altissimo, 2016; Tubaro et al., 2016; Van Waes and Van den Bossche, 2020). Participants (egos) were asked to name people they believed to belong to their social surroundings (alters) and to specify the relations between these individuals. In addition, the young women were asked to rate their alters’ significance in their life and put them into three concentric circles according to this ranking, with the ego at the center (Figure 1). Alters subjectively considered to be more significant to ego are in the closest circle to her (1-rated) and those alters who were not included in these circles were considered by the young women as not significant (4-rated). All the young women completed informed consent forms. The research complies with ethical norms of [the University name was removed for review purposes] University and was approved by the IRB.

In this paper we will focus on personal networks in which the ego is the female blogger with anorexia, the alters are the members of the perceived social surroundings, and the ties between these people are the social relations (talking and other forms of interaction) that, according to the ego’s suggestions, exist between her and her alters.

### Social Network Analysis: Measures

One of the central network characteristics is the *network size*. The sizes of network structures (graph) can be captured by calculating the number of nodes and edges. Within the framework of this study, we considered the network size to be the number of alters (nodes) connected (edges) to the ego (target node).

As a measure of social cohesion, we used the proportion of *isolates* in a given network. An isolate is an actor who does not have any connections within the network. The proportion of isolates is the ratio between the number of isolates and the network size.

We used Python 3.7.4 for the computations, with networkx Python package 2.6.2 employed to calculate the descriptive statistics of the network. Additionally, we used Pearson’s correlation coefficient in and Student’s *t*-test (SciPy 1.7.1 package).

## Results

**FIGURE 2:**
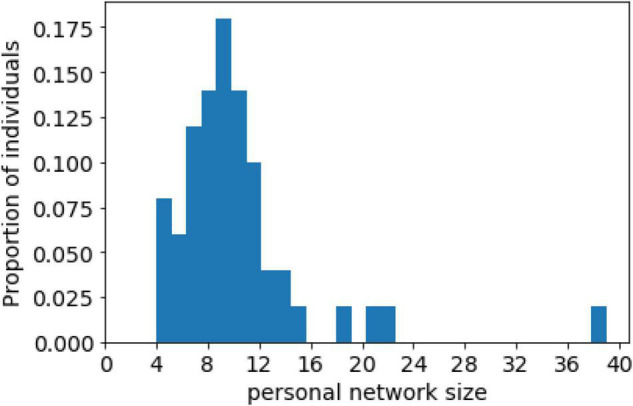
Distribution of personal network size (No networks had fewer than four members).

The distribution of the personal network size is presented in Figure 2. The network size varied from 4 to 39. For the majority of the individuals (*N* = 47, 94% of the sample) it ranged between 5 and 16. On average, the number of alters was 10.24 (SD = 5.56); the median was 9. The distribution of the network size was not normal. We found a small proportion of outliers with extremely high numbers of alters, which aligns with network theory (Barabási and Albert, 1999), which predicts the presence of multiple actors with limited numbers of ties and a few individuals with many ties (so-called hubs). After the exclusion of the most popular individual (with 39 alters in their personal network), the average number of alters for the sample decreased to 9.65 (SD = 3.74). The median network size remained at 9. In further calculations, we analyzed the sample without this outlier.

Our findings support H1, in that the network size for the major part of young women with anorexia ranges from 5 to 16.

**FIGURE 3:**
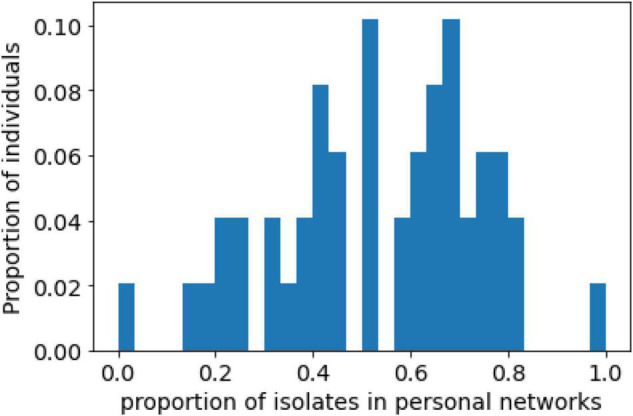
Distribution of the proportion of isolates in personal networks.

On average, there were 5.33 (SD = 3.33) isolated individuals in the observed personal networks, with a median of 5. The proportion of isolated individuals in personal networks was 0.53 (SD = 0.21) (Figure 3). These results support H2. These values might suggest that people tend to diversify their social relationships and maintain connections with both cohesive communities and isolated individuals. Approximately half of a given personal network consisted of isolated alters, while the rest of the network was a connected group of multiple cohesive communities.

**FIGURE 4:**
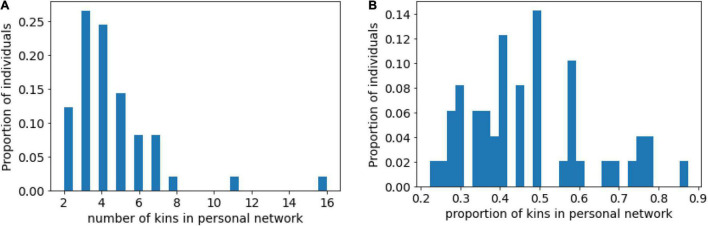
**(A)** Distribution of number of kin alters in personal networks; **(B)** Distribution of proportion of kin alters in personal networks.

On average, the individuals maintained relationships with 4.51 family members (SD = 2.47) (Figure 4A). For each network we computed the proportion of kin alters in personal networks. For the whole sample, the average fraction of kin alters was 0.47 (SD = 0.16) (Figure 4B). Thus, we conclude that the proportion of kin in personal networks of women with anorexia is not greater than the proportion of non-kin, in contradiction to H3.

**FIGURE 5:**
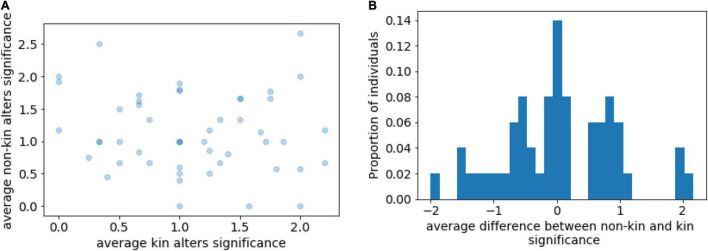
**(A)** Relationship between average kin and non-kin alter significance; **(B)** Distribution of average difference between non-kin and kin alter significance.

To examine whether young women with anorexia tend to select non-kin members as the closest alters in their networks, for each personal network, we computed the average significance of the kin and non-kin members in the personal network (Figures 5A,B). The average significance of kin alters was 1.13 (SD = 0.59), and 1.12 (SD = 0.60) for non-kin alters (*p*-value = 0.95, *t*-test). Thus, we did not find a difference between the levels of significance for kin and non-kin alters, and the results do not support H4.

**FIGURE 6:**
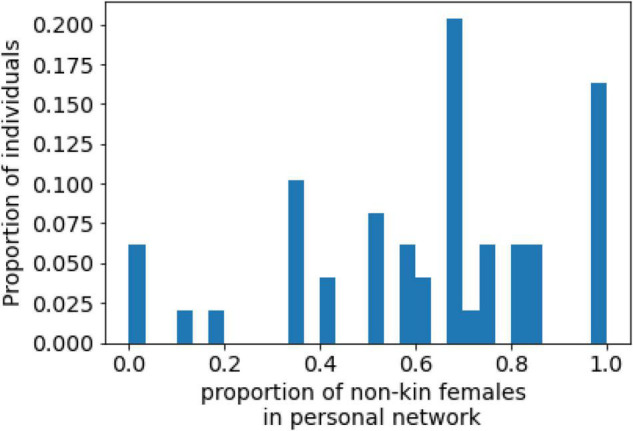
Distribution of the proportion of non-kin female alters.

The average proportion of females in non-kin networks was 0.62 (SD = 0.27), and the median was 0.67 (Figure 6). In more than two thirds of the participants’ networks (68%), the proportion of females among the non-kin alters was greater than half. Thus, the evidence supports H5, and we might conclude that participants tend to create ties with females, and that this tendency is independent of their overall network structure.

Additional descriptive statistics on personal networks of young women with anorexia and association between the variables are reported in the Supplementary Appendix.

## Discussion

This paper addresses the gap in the literature on the relationships of young women with anorexia by providing information on the quantitative aspects of their personal networks. Previous studies on this topic are scarce (Quiles Marcos and Terol Cantero, 2009; Tubaro and Mounier, 2014; Pallotti et al., 2018), and most is qualitative, which significantly limits scholars’ and practitioners’ abilities to develop reliable measures for interventions and treatment outcomes based on network indicators. We report on young women’s personal networks in terms of size, structure, and kinship network. The calculations of these parameters were made on the basis of 50 ego networks described by Russian-speaking bloggers in qualitative interviews in the summer of 2020.

The results regarding the average personal network size of young women with anorexia largely agree with the previous research (H1). We demonstrated, consistent with Quiles Marcos and Terol Cantero (2009), Tubaro and Mounier (2014), and Pallotti et al. (2018) that on average, these women maintain relationships with 10 people. A personal network of this size does not, at first, appear critically small because studies show that the active network of a person in the general population usually comprises at least 20–30 people (Maguire, 1983; Lubbers et al., 2019). However, these numbers include both online and offline contacts. The literature on the utilization of mental health services demonstrates that a limited number of personal connections is often related to increased usage of mental health services (Mitchell, 1989; Albert et al., 1998; Thoits, 2011) because the person is not surrounded by people who can provide the necessary care and support. Moreover, small personal network size is associated with an elevated likelihood of compulsory hospital admission (Kogstad et al., 2013). Therefore, we suppose that in future studies it would be important to address the association between personal network size and the severity of anorexia outcomes for women with this condition. This could extend understanding of whether interventions tailored toward the improvement of the general social skills of young women (Spence, 2003) are also effective in the prevention of severe anorexia symptoms (Pratt and Woolfenden, 2002; Dimitropoulos et al., 2016; Hay et al., 2019; Gan et al., 2021).

In line with previous research, we found that our participants had connections with both cohesive communities and isolated individual actors (H2) (Tubaro and Mounier, 2014; Pallotti et al., 2018). By contrast, in the general population, a person’s social network is made up of 10 percent or less of isolated alters on average (Golinelli et al., 2010; Martí et al., 2017; Stadel and Stulp, 2022). This means that the social network of the ordinary person is much denser than the networks of our research participants. The connection between social network density and diversity is complicated (Walker, 2015; Lee et al., 2020). Network density is positively related to personal wellbeing only when the individual is in a self-affirming social environment (Walker, 2015). Otherwise, being surrounded by many people who actively interact with each other does not have a positive effect on the individual’s mental health because she feels trapped in the social groups in which she is included. Network diversity, despite being connected to improvements in psychological wellbeing, is not related to an increase in personal feelings of social support (Müller et al., 2007). This means that in a woman with anorexia, a high level of social contact diversity could coincide with personal feelings of loneliness. We believe this could be true of our study participants, who told us during the interviews that many people they interact with tend to produce negative body talk and collaborate on this production of negative statements (Mikhaylova, 2022). Young women often cannot cease communication with these abusive individuals because they are their classmates, teachers, or even family members (Shannon and Mills, 2015). Furthermore, our data do not allow us to quantitatively determine the way the diversity and density of the personal social networks of women with different incomes are connected to their experience of anorexia, which is a question that future studies can address. Economic inequality means that it might be easier for some women to change their social environments and, as a result, improve their psychological wellbeing; for others, such a move may be much more problematic.

Contrary to our assumptions based on previous studies (Quiles Marcos and Terol Cantero, 2009; Tubaro and Mounier, 2014; Pallotti et al., 2018), we found that the proportion of kin members in the participants’ personal networks was not higher than the proportion of other members of these networks (H3). Furthermore, people in the general population also report that almost half of their personal network consists of relatives, and this figure holds across different country samples (Wellman and Wortley, 1989; Dunbar and Spoors, 1995; Grossetti, 2007). Because the quality of family relationships could influence the success of psychotherapy (Sapin et al., 2016; Fleming et al., 2021), we suggest cautious interpretations of the proportion of kin in the personal networks of people with psychological problems. Women with mental health problems who experience overload and ego-centered conflict in family relationships could show patterns of evaluated psychological distress (Sapin et al., 2016; Tournier et al., 2021). Therefore, we believe that future studies, preferably longitudinal, are necessary to clarify the connection between kinship networks and treatment outcomes for women with anorexia.

We found no statistically significant difference between the subjective closeness of kin and non-kin members of the participants’ social networks (H4). This contradicts the assumptions formulated on the basis of the studies by Brotsky and Giles (2007), Haas et al. (2011), and Pallotti et al. (2018). Moreover, this contradicts the research on the general population, which claims that people at various life stages tend to perceive their relatives as socially closer than non-kin members of their personal networks (Shulman, 1975; Wellman and Wortley, 1989; Aeby et al., 2018). Nevertheless, we deduced that women with larger personal networks maintain both deep and shallow relationships with non-kin members of their networks. Support from family members has been described in the literature as being more important for one’s personal mental health than support from friends and significant others (Shor et al., 2013; Aeby et al., 2020). Nonetheless, as the importance of familial support increases with age (Shor et al., 2013; Widmer et al., 2018; Woods et al., 2020), we believe it likely that the value of family support for older women (Gadalla, 2008) with anorexia might be higher. This idea would need further investigation carried out on older individuals.

In accordance with Tubaro and Mounier (2014) and Pallotti et al. (2018), we discovered that young women with anorexia had a high proportion of female non-kin personal network members (H5). This finding corresponds with social comparison studies that have shown that body dissatisfaction and eating problems among women are related to the internalization of the body-related attitudes shared by significant women in their lives, such as mothers, sisters, and close female friends (Thompson et al., 1999; Lev-Ari et al., 2014a,b; Nerini et al., 2016; Betz et al., 2019; Pollet et al., 2021). Additionally, this result is in line with research on the general population, which has found that non-kin contacts account for more than half of people’s personal networks (Dunbar and Spoors, 1995; McPherson et al., 2006; Roberts et al., 2008, 2009). Because our participants came from Russian-speaking countries, we acknowledge that gender ideology and social expectations of women in this cultural context (White, 2005; Barrett and Buckley, 2009; Kosterina, 2012; Turbine, 2012) could influence the functioning of social comparison mechanisms among women, especially in the family context. Therefore, we think that comparative research of the personal networks of women with anorexia from different regions worldwide is needed to show how the environments of the state and social institutions can moderate the effect of social connections on the wellbeing of such women.

Because the data was collected during a COVID-19 lockdown, participants were additionally asked about the perceived effects of the pandemic on their mental and physical health and social networks. They mentioned that lockdown and other consequences of the COVID-19 pandemic have influenced their eating behavior. As has been reported in comparable research (Phillipou et al., 2020; Schlegl et al., 2020), participants claimed that they started to exercise and control their food intake more. They associate these changes with lockdown restrictions. Women also reported that they felt more anxious about their current and future educational and career prospects, which corresponds with studies of emotional wellbeing during COVID-19 pandemic among people with eating disorders (Sideli et al., 2021; Linardon et al., 2022). These studies have demonstrated that during COVID-19 pandemic people with eating disorders have experienced elevated feelings of stress, fear, and anxiety. At the same time, our study participants did not note any change in their relations with people in their personal networks contrary to some of the other studies of the perceived social support among people with eating disorders during COVID-19 pandemic (Sideli et al., 2021; Linardon et al., 2022). Perhaps our participants were able to maintain relationships with people from their personal networks *via* digital technologies that is why they did not notice any changes in their social networks. Additionally, they could have felt peer support from the members of the online eating disorder communities as many researchers, for example, Albano et al. (2021) have discovered that during emergent global situations such as COVID-19 pandemic these communities may provide for the members the feelings of being understood by people with comparable life situations.

## Limitations

Our research is not without shortcomings. First, the sample comprises young white women, whose personal networks may differ from those of women of color (Ajrouch and Antonucci, 2018) and those who belong to other minorities (Frost et al., 2016; Watson et al., 2019; Fischer, 2021), as well as those of women with anorexia who belong to other age groups (Midlarsky and Nitzburg, 2008; Lapid et al., 2010; Scholtz et al., 2010). Second, because women with anorexia are a hard-to-reach population, especially in Russian-speaking countries, where there is no officially gathered data on the prevalence of EDs, in this paper, we estimated the network characteristics of only 50 women. However, we hope that future studies can utilize larger samples, creating opportunities for a wider range of between-group comparisons. Third, our sample comprises women bloggers and we do not know whether their social contacts differ from those of young women with anorexia who do not blog. Fourth, reports on the personal relations that young women maintain should be regarded with caution due to memory (Brewer, 2000), sensitivity (Cronin et al., 2020), problems with the attribution of roles to the members of personal networks (Bush et al., 2017), and other interview-related issues (Feld and Carter, 2002; Kogovšek and Ferligoj, 2005). Fifth, when reporting on the personal networks of young women based on their narratives, we should remember that these are only the young women’s perceptions of their relations (Bayer et al., 2020; Feld and McGail, 2020). Additional research is needed that includes the perspectives of the members of these personal networks (Suitor et al., 2020) to reveal how the members of these personal social circles perceive their relations with young women with anorexia.

## Conclusion

Our study demonstrates that young women with anorexia do have small personal social networks. On average, half of the alters in their personal networks are in communication with each other and potentially might be involved in the same social circles. We did not find that kin alters outnumber non-kin in these social networks. At the same time, it could be argued that women with anorexia maintain relationships primarily with other women. Further research, better on larger samples, is needed to elucidate whether these personal network characteristics are similar between women of different ages, incomes, ethnicities, and cultural groups.

## Data Availability Statement

The raw data supporting the conclusions of this article will be made available by the authors, without undue reservation.

## Ethics Statement

The studies involving human participants were reviewed and approved by HSE University. The patients/participants provided their written informed consent to participate in this study.

## Author Contributions

Both authors listed have made a substantial, direct, and intellectual contribution to the work, and approved it for publication.

## Conflict of Interest

The authors declare that the research was conducted in the absence of any commercial or financial relationships that could be construed as a potential conflict of interest.

## Publisher’s Note

All claims expressed in this article are solely those of the authors and do not necessarily represent those of their affiliated organizations, or those of the publisher, the editors and the reviewers. Any product that may be evaluated in this article, or claim that may be made by its manufacturer, is not guaranteed or endorsed by the publisher.

## Footnotes

1 This dataset is a part of one of the authors’ Ph.D. dissertations and can be obtained from the author directly if needed.
