# Supplementary material for: Anorexia and Young Womens’ Personal Networks: Size, Structure, and Kinship
Source: Front Psychol. 2022 Apr 19;13:848774. doi: 10.3389/fpsyg.2022.848774 (PMC9063839; doi:10.3389/fpsyg.2022.848774)
Supplement: Supplementary file 1 [file Table_1.DOCX]

**Appendix**

***Sample description***

Table 1. Sample description

| Participant number | Age | Place of residence |
| --- | --- | --- |
| 1. | 17 | Ufa |
| 2. | 19 | Saint Petersburg |
| 3. | 20 | Almaty |
| 4. | 19 | Izhevsk |
| 5. | 17 | Rostov-on-Don |
| 6. | 17 | Moscow |
| 7. | 15 | Slobodskoy |
| 8. | 17 | Surgut |
| 9. | 20 | Ryazan |
| 10. | 17 | Ekaterinburg |
| 11. | 15 | Gubkin |
| 12. | 16 | Surgut |
| 13. | 16 | Saint Petersburg |
| 14. | 19 | Nahodka |
| 15. | 18 | Moscow region |
| 16. | 18 | Balashov |
| 17. | 16 | Ryazan |
| 18. | 19 | Tymen |
| 19. | 25 | Paris |
| 20. | 24 | Kiev |
| 21. | 22 | Saint Petersburg |
| 22. | 15 | Tomsk |
| 23. | 20 | Moscow |
| 24. | 23 | Moscow |
| 25. | 19 | Moscow |
| 26. | 17 | Kirov |
| 27. | 17 | Nizhny Tagil |
| 28. | 16 | Nizhny Tagil |
| 29. | 17 | Surgut |
| 30. | 17 | Pskov |
| 31. | 18 | Barnaul |
| 32. | 17 | Irkutsk |
| 33. | 17 | Saint Petersburg |
| 34. | 17 | Rostov-on-Don |
| 35. | 14 | Rasskazovo |
| 36. | 19 | Voronezh |
| 37. | 14 | Saransk |
| 38. | 20 | Murmansk |
| 39. | 14 | Ufa |
| 40. | 17 | Barnaul |
| 41. | 18 | Saint Petersburg |
| 42. | 17 | Arkhangelsk |
| 43. | 15 | Tolyatti |
| 44. | 17 | Novokuznetsk |
| 45. | 20 | Omsk |
| 46. | 24 | Minsk |
| 47. | 20 | Saint Petersburg |
| 48. | 15 | Vladivostok |
| 49. | 22 | Saint Petersburg |
| 50. | 17 | Moscow |

***Descriptive statistics on the social networks of young women with anorexia***

In this part of the appendix we report the structural properties of analyzed social networks. We use the following social network metrics:

*Average degree centrality*. Degree centrality is the number of nodes connected to the target node (Wasserman & Faust, 1994). The average degree centrality corresponds to the mean degree of centrality in a given network.

*Transitivity.* Social network theorists argue that many of the most interesting and basic questions of social structure arise with regard to triads (Wasserman & Faust, Hanneman & Riddle, 2005). One of the most prominent characteristics of triads is the transitivity index. It means that we count the number of times that, if we see AB and BC, we also see AC (Hanneman & Riddle, 2005). Simply put, transitivity reflects the proportion of close triads in the social network. In most empirical networks transitivity values are large (up to 0.5), which means that individuals tend to create groups of at least three connected individuals.

We also examine the presence of *communities* within the social networks. The generally accepted way of quantifying the existence of communities within a given network is by using the *modularity* index. The modularity is, up to a multiplicative constant, the number of edges falling within groups minus the expected number in an equivalent network with edges placed at random (Newman 2006). It can be either positive or negative, with positive values showing the presence of cohesive subgroup structures. Newman argues that in the majority of real-world social networks, modularity varies from 0.2 to 0.7, exhibiting the presence of group structures. The descriptive statistics on these metrics are in Table 1S; the distributions of these measures are on Figures S1-S3.

Table S1. Descriptive statistics on the personal social networks of young women with anorexia

| Parameter | Mean | Median | St. dev. |
| --- | --- | --- | --- |
| Average degree | 2.75 | 2.71 | 0.74 |
| Transitivity | 0.40 | 0.36 | 0.23 |
| Modularity | 0.17 | 0.17 | 0.07 |


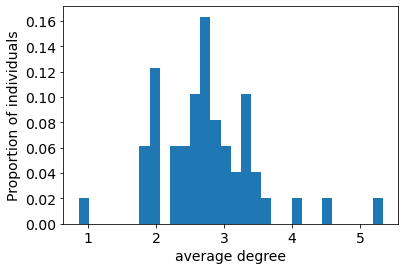


*Figure S1.* Average degree distribution.


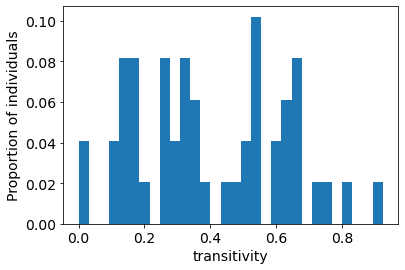


*Figure S2.* Transitivity distribution.


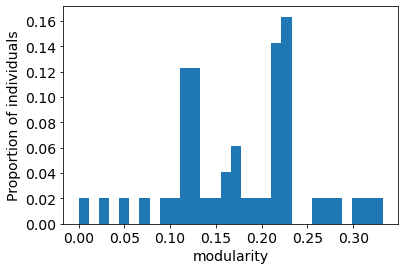


*Figure S3.* Modularity distribution.

The summary of the empirical finding follows. First of all, our computations show that on average, alters in personal networks of women with anorexia support 2.75 ties. Secondly, it was discovered that young women with anorexia create social networks in which alters on average support 2.75 connections with each other. Thirdly, we found out that these networks demonstrate medium transitivity, which means that alters of the personal networks are associated not only in dyads, but also in triangles and larger social groups. Finally, it was detected that the modularity index of these networks is moderate, which might suggest that women with anorexia do not form connections with multiple distinct communities.
